# Inflammatory Metabolic Index and Metabolic-Inflammatory Stress Index as New Biomarkers for Complicated and Perforated Acute Appendicitis

**DOI:** 10.3390/jcm14155281

**Published:** 2025-07-25

**Authors:** Sidere M. Zorrilla-Alfaro, Nestor A. Lechuga-Garcia, Arturo Araujo-Conejo, Leticia A. Ramirez-Hernandez, Idalia Garza-Veloz, Alejandro Mauricio-Gonzalez, Ivan Delgado-Enciso, Iram P. Rodriguez-Sanchez, Margarita L. Martinez-Fierro

**Affiliations:** 1 Molecular Medicine Laboratory, Unidad Académica de Medicina Humana y C.S, Universidad Autónoma de Zacatecas, Zacatecas 98160, Mexico; siderezorrilla@gmail.com (S.M.Z.-A.); idaliagv@uaz.edu.mx (I.G.-V.); amgdark@uaz.edu.mx (A.M.-G.); 2 Hospital General Zacatecas “Luz González Cosío”, Servicios Públicos de Salud IMSS-BIENESTAR, Zacatecas 98160, Mexico; drnestorlechuga@gmail.com (N.A.L.-G.); aaraujo2002@yahoo.com (A.A.-C.); 3 Unidad Académica de Matemáticas, Universidad Autónoma de Zacatecas, Paseo la Bufa, Av. Solidaridad, Zacatecas 98066, Mexico; lramirez@uaz.edu.mx; 4 School of Medicine, University of Colima, Colima 28040, Mexico; ivan_delgado_enciso@ucol.mx; 5 State Cancerology Institute of Colima, Health Services of the Mexican Social Security Institute for Welfare (IMSS-BIENESTAR), Colima 28085, Mexico; 6 Laboratorio de Fisiologia Molecular y Estructural, Facultad de Ciencias Biológicas, Universidad Autónoma de Nuevo León, San Nicolás de los Garza 66455, Mexico; iramrodriguez@gmail.com

**Keywords:** appendicitis, biomarker, diagnosis, glucose, lymphocyte, creatinine

## Abstract

**Background**: Acute appendicitis is a common emergency requiring abdominal surgery. Despite its prevalence, there are no specific biomarkers for its diagnosis and prognosis. The aim of this study was to assess the basic laboratory tests of patients with acute appendicitis and to evaluate and integrate biochemical variables into the diagnosis of appendicitis. **Methods**: This was a retrospective, cross-sectional cohort study that included data from patients who underwent an appendectomy. Two groups of patients were considered based on their surgical (non-complicated/complicated appendicitis) or pathological diagnosis (non-perforated/perforated appendicitis). Factor analysis was carried out to identify communalities to put forward classificatory indices. Receiver operating characteristic (ROC) analysis was used to assess the accuracy of the predictions. **Results**: The cohort included 246 patients (51.6% male, mean age: 24.79 ± 19.32 years). By using their biochemical data, we generated 6 new indices whose areas under the ROC curve (AUC) ranged between 0.632 and 0.762 for complicated appendicitis and from 0.597 to 0.742 for perforated appendicitis. Inflammatory Metabolic Index (IMI) at the fixed cutoffs was a promising biomarker for both histopathological and surgical diagnoses with odds ratios (OR) of 10.45 and 5.21, respectively. The Metabolic-Inflammatory Stress Index (MISI) showed high specificity (over 72%) and significant AUC values for both diagnoses (0.742 and 0.676). These findings were reinforced by significant *p*-values and Youden indices. **Conclusions**: IMI and MISI were demonstrated to be effective biomarkers for complicated and perforated appendicitis. IMI provides predictive capability, while MISI offers specificity and significant AUC values for both histopathological and surgical diagnoses. Incorporating these biomarkers could enhance the accuracy of appendicitis diagnosis and potentially guide clinical decision-making.

## 1. Introduction

Acute appendicitis is defined as inflammation of the vermiform appendix [1]. It is the most common abdominal surgical emergency in the world, with an annual incidence of 96.5 to 100 cases per 100,000 adults [2] and 1 per 1000 individuals in Mexico [3].

**Figure 1:**
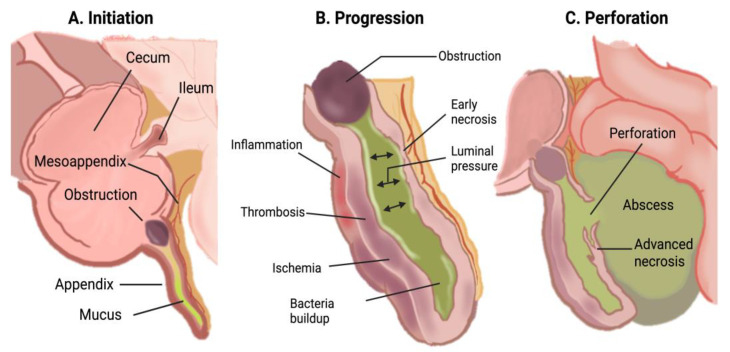
Pathophysiology of acute appendicitis [2].

The underlying pathophysiology of acute appendicitis is attributed to the obstruction of the lumen of the cecal appendix by various factors. These include appendicoliths (fecaliths), calculi, lymphoid hyperplasia, infections, and benign or malignant tumors, all of which can lead to mucus accumulation. Less common causes, particularly in pediatric patients, include foreign bodies [4] and parasitic infections such as *Enterobius vermicularis* [5]. The initial increase in intraluminal pressure precipitates thrombosis of small vessels and lymphatic stasis. These events result in ischemia and bacterial overgrowth, which may progress to abscess formation. If the necrotic wall becomes perforated, the contents are released into the peritoneal cavity, potentially resulting in phlegmon or generalized peritonitis (Figure 1) [2,6]. The attachment of the appendix to the cecum is constant; the tip may migrate to various positions, including retrocecal, subcecal, preileal, and pelvic [7]. These variations may complicate the diagnosis of acute appendicitis, as the site of pain and clinical examination findings will reflect the anatomic position of the appendix [8] and become relevant because the risk of perforation is directly related to the time from the onset of symptoms to diagnosis and surgical intervention [1].

The management of appendicitis typically involves an appendectomy, a procedure with a generally low-risk surgical profile. The outcomes of this surgery, such as the morbidity and mortality rates of patients who have undergone appendectomy, are strongly influenced by the severity of the appendicitis and the presence of comorbidities [6]. Interestingly, while the overall incidence of acute appendicitis has been on the decline, cases of perforated appendicitis have seen an uptick [9]. The lifetime risk of acute appendicitis is slightly higher in males than in females (8.6% vs. 6.7%). However, females have a higher lifetime risk of undergoing an appendectomy (23.1% vs. 12.0%) [6,10]. Men are more likely to experience perforated appendicitis than women, with rates of 31 per 100,000 person-years for men compared to 25 per 100,000 person-years for women [2,11].

The standard diagnostic approach for appendicitis includes a review of the clinical history, physical examination, laboratory tests, and potentially imaging if the diagnosis remains uncertain [2]. Although the attachment of the appendix to the cecum is constant, the tip may migrate to various positions, including retrocecal, subcecal, preileal, and pelvic [7]. These variations may complicate the diagnosis of acute appendicitis, as the site of pain and clinical examination findings will reflect the anatomic position of the appendix [8], and become relevant because the risk of perforation is directly related to the time from the onset of symptoms to diagnosis and surgical intervention [1]. The definitive diagnosis of acute appendicitis is typically confirmed through histological examination post-appendectomy [12]. Delayed diagnosis of complicated appendicitis is associated with significant morbidity and mortality [13]. A cohort study by Harris and colleagues highlighted discrepancies between intra-operative findings and histopathological diagnoses of acute appendicitis, underlining the challenges in accurately diagnosing acute appendicitis [14]. Prompt and precise diagnosis can significantly decrease the risk of perforation and other serious complications associated with this condition [1]. The sensitivity and specificity of an elevated white blood cell count in acute appendicitis range approximately from 70% to 80% and from 55% to 65%, respectively [15]. In the same way, a diminished lymphocyte count has been identified as a stress indicator, with lymphopenia linked to appendicitis [16]. The concentration of proinflammatory cytokines closely correlates with both the absolute neutrophil and the absolute lymphocyte counts, as reflected by the neutrophil-lymphocyte ratio (NLR) [17]. Prior research has established a significant association between mean platelet volume, NLR, and platelet-lymphocyte ratio (PLR) with inflammatory processes [18,19,20]. These hematological indices have been scrutinized in acute appendicitis cases [16,19,20,21,22,23,24,25,26]. The systemic immune inflammation index (ISS), which integrates counts of platelets, neutrophils, and lymphocytes (platelet × neutrophil/lymphocyte), emerges as a promising biomarker with potentially greater sensitivity and specificity than both INL and IPL [20,27,28].

In the efforts to contribute to the diagnosis, several clinical scales have been developed. Among these scales are the Alvarado scale [29], Pediatric Appendicitis Scale [30], and Inflammatory Response to Appendicitis scale [6]. These scales include only variables related to immune cells or inflammatory-related markers such as C-reactive protein [1,2,6,31]; however, other important biochemical parameters involved in the appendicitis pathophysiology have not been considered. It is well known that acute appendicitis can cause stress, activating the hypothalamic-pituitary-adrenal (HPA) axis and resulting in increased secretion of various hormones, such as cortisol, norepinephrine, epinephrine, glucagon, and growth hormone [14,32]. These hormonal shifts drive metabolic changes: cortisol increases hepatic gluconeogenesis and antagonizes insulin, leading to hyperglycemia [32,33]. This state of stress-induced hyperglycemia enhances immune cell function, as elevated glucose levels support the energetic demands of neutrophils and lymphocytes engaged in the inflammatory response. Concurrently, cortisol has anti-inflammatory effects by stabilizing lysosomal membranes, reducing capillary permeability, and decreasing leukocyte migration to the inflamed area and lymphocyte proliferation [32,33,34]. These processes underscore the dual role of cortisol in modulating both metabolic and immune profiles during acute appendicitis, where it helps to manage inflammation and maintain blood glucose levels, although it may also contribute to insulin resistance and hyperglycemia when combined with other counter-regulatory hormones [32,33]. According to the above, it is reasonable to consider that these active processes may be manifested biochemically at a systemic level and could assist or contribute to the diagnosis of appendicitis. Considering that there is currently no specific biomarker available for the diagnosis and prognosis of acute appendicitis, the aim of this study was to evaluate the basic laboratory tests of patients with acute appendicitis and integrate biochemical variables into the diagnosis of appendicitis. The diagnostic/prognosis values of these variables were also compared with existing indices.

## 2. Materials and Methods

### 2.1. Study Population and Study Design

**Figure 2:**
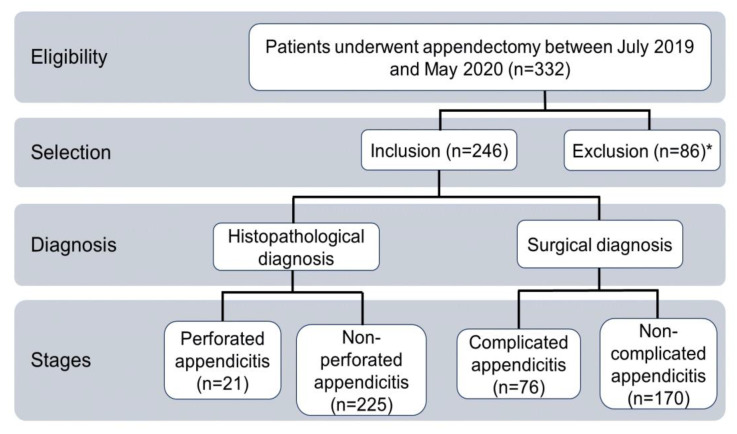
Flow chart of the inclusion of study participants. * Three patients excluded because of incidental or secondary appendicectomies; Eighty-three patients excluded because of data loss.

This was a retrospective, cross-sectional cohort study that included data from 332 patients who underwent an appendectomy between January 2019 and May 2020, regardless of age and sex (Figure 2). Patients with incomplete laboratory or histopathological records and those with incidental or secondary appendectomies were excluded. According to above, the study included 246 patients. Two groups of patients were considered, each containing two subgroups classified according to their histopathological [35] or surgical [31] diagnoses. The personal, epidemiological, and clinical data were collected from medical records, and they included sex, age, days of hospital stay, laboratory results upon admission to the emergency department, surgical diagnosis, and histopathological diagnosis.

### 2.2. Definitions and Criteria for Surgical and Histopathological Diagnosis

In this study, the classification of the participants was based on their histopathological and surgical findings. For the histopathological diagnosis, patients were classified into non-perforated appendicitis (edematous, suppurative, gangrenous) and perforated appendicitis. ‘Edematous’ refers to inflammation of the serosa and subserosa, where the infiltrate does not extend beyond the outer muscularis propria; ‘Suppurative’ is characterized by neutrophilic infiltration of the mucosa, submucosa, and muscularis propria, transmural inflammation, extensive ulceration, and intramural abscesses with vascular thrombosis; ‘Gangrenous’ involves inflammation that affects the entire thickness of the affected tissue, resulting in areas of tissue death (necrotic areas) and widespread damage to the mucosal lining (mucosal ulceration); Finally, ‘perforation’ refers to the loss of continuity in the wall [35,36]. For surgical diagnoses, we categorized them into uncomplicated appendicitis (stage I and II) and complicated appendicitis (stage III and IV), following the guidelines established by the European Association for Endoscopic Surgery in their 2015 consensus meeting [31]. This classification allows for standardized stratification of appendicitis severity during surgery: Stage I (inflamed but non-complicated appendix), stage II (phlegmonous or suppurative), stage III (gangrenous), and stage IV (perforated).

### 2.3. Assessment of Indices and Ratios

The NLR and PLR were calculated by dividing the *neutrophil count* by the *total lymphocyte count* and the *platelet count* by the *total lymphocyte count*, respectively. SII involved multiplying the quotient of the *neutrophil count* and *total lymphocyte count* by the *platelet count*.

Based on the performed multivariate analyses, we put forward and evaluated several indices and ratios as shown in Table 1.

### 2.4. Statistical Analysis

Descriptive statistics, measures of central tendency, and frequency tables were utilized. Chi-Square/Fisher tests were performed for nominal variables, and *t*-Student/U Mann–Whitney tests were performed for continuous variables. One-Way Analysis of Variance or Kruskal–Wallis One-Way Analysis on Ranks, as appropriate, was performed to analyze the surgical and histopathologic diagnostic phases and stages. Factor analysis was carried out to identify groups of variables related to acute appendicitis. This analysis included all the variables with significant results from the comparisons between study groups, considering histopathological and/or surgical diagnoses. In addition, the calculated values for the NRL and PLR ratios and the SII index were also considered (Section 2.3). From those parameters, correlation matrices were generated to group these variables into components that explained the cases of acute appendicitis. The models were selected considering the KMO (Kaiser–Meyer–Olkin value), which is a measure of sampling adequacy used in factor analysis. Models with a KMO value greater than 0.6 were considered as suitable for factor analysis. After, these models were considered to construct ratios and/or indices with the most representative variables. Factor analysis was carried out using SPSS 29.0.2.2. Receiver operating characteristic curves (ROC) were constructed and used to calculate areas under the ROC curve (AUC) and the cut-off points for each index and ratio. Sensitivity, specificity, positive and negative predictive values, and the Youden index were obtained. Odds Ratio (OR) values were also calculated for significant variables at the fixed cutoff for each index. Post-hoc power analysis was conducted to evaluate the adequacy of the sample size used. Using the observed ORs, group sizes, and a two-tailed significance level of 0.05, the statistical power was calculated for each index. To obtain these estimates, the ORs obtained were converted into approximate standardized effect sizes (Cohen’s d) using the commonly accepted transformation $d=\ln\left(OR\right)\frac{\sqrt{3}}{\pi }$. This approach allowed to assess power using a normal approximation model for differences between independent groups.

Univariate and ROC analyses were performed using Sigma Plot software version 14.5. A statistically significant value of *p* = 0.05 was used with 95% confidence intervals.

## 3. Results

### 3.1. General Characteristics and Clinical Parameters of the Study Population

Two hundred and forty-six patients participated in the study, with 51.6% being male and 48.4% being female. The age range was from 1 to 91 years old, with a median of 18.5 and a mean of 24.79 ± 19.32. Most appendectomies, 96.75%, were performed via the conventional open technique, whereas the laparoscopic technique was utilized in 3.25% of the cases. Incision types varied, with median incisions accounting for 28.86%, Rocky Davis 37.4%, McBurney 15.86%, Battle incisions 14.63%, and laparoscopic incisions 3.25%. In terms of appendicular stump closure, the Pouchet technique was applied in 76.83% of cases, followed by the Parker Kerr method in 7.32%, Halsted technique in 2.84%, and the Zuckerman method in 13.01%.

**Figure 3:**
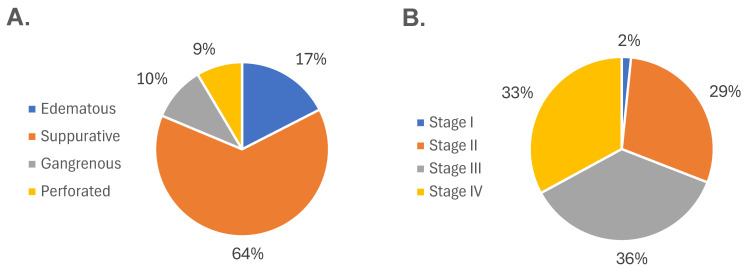
Distribution of participants according to disease severity. (**A**) Histopathological classification of acute appendicitis, showing the proportion of cases diagnosed as edematous, suppurative, gangrenous, and perforated. (**B**) Surgical classification based on intraoperative findings, categorized into stages I through IV according to increasing severity. Both classification systems demonstrate a progressive distribution of cases across the clinical spectrum of acute appendicitis.

Figure 3 shows the distribution of participants according to their histopathological and surgical diagnoses. Based on histopathological evaluation, 43 (17.48%), 157 (63.82%), 25 (10.16%), and 21 (8.54%) patients were diagnosed with edematous, suppurative, gangrenous, and perforated appendicitis, respectively (Figure 3A). Regarding surgical staging, stage I accounted for 4 (1.63%) cases, stage II for 72 (29.27%), stage III for 89 (36.18%), and stage IV for 80 (32.92%) cases (Figure 3B). Overall, significant differences in clinical and laboratory parameters were observed across both histopathological and surgical classifications. These included age, leukocyte and lymphocyte counts, neutrophil and lymphocyte percentages, and coagulation markers (prothrombin time, INR, and partial thromboplastin time), as well as glucose, serum creatinine, and chloride levels (*p* < 0.05).

### 3.2. Univariate Data Modelling to Identify Predictors Related to Appendicitis Outcome: Uncomplicated/Complicated and Non-Perforated/Perforated

With the aim of identifying variables associated with the appendicitis outcome, the patients were classified based on their surgical and histopathological diagnosis. Specifically, during the statistical approximation, the surgical staging was categorized as uncomplicated appendicitis (stage I and II) and complicated appendicitis (stage III and IV) [31]. According to their histopathological diagnosis, the patients were classified as those with non-perforated appendicitis (edematous, suppurative, and gangrenous) and perforated appendicitis (Section 2). The results are presented accordingly (Table 2).

There was no significant difference in sex distribution between cases with perforated (4.47% male, 4.06% female) and non-perforated (47.16% male, 44.31% female) appendicitis (*p* = 0.199). However, there was a significant difference in mean age between the two groups, with perforated cases having a higher mean age (36.67 ± 24.58) compared to non-perforated cases (23.68 ± 18.43) (*p* = 0.011). In addition, the mean number of days of hospital stay was longer for perforated appendicitis (4.71 ± 3.64) compared to non-perforated appendicitis (3.10 ± 2.75) (*p* = 0.079), as presented in Table 2. The group of patients with perforated appendicitis showed a significant decrease in the percentage of lymphocytes compared to those with non-perforated appendicitis (*p* ≤ 0.001). Additionally, higher values were observed for prothrombin time (*p* = 0.02), INR (*p* = 0.018), and partial thromboplastin time (*p* = 0.008), as well as increased levels of glucose (*p* = 0.001) and serum creatinine (*p* = 0.003) (Table 2).

The mean hospital stay was 3.46 ± 2.74 in the group of complicated appendicitis and 2.45 ± 2.96 in the group of uncomplicated appendicitis (*p* ≤ 0.001). In the group of patients with complicated appendicitis, there was a significant decrease in the percentage of lymphocytes (*p* ≤ 0.001) and lymphocyte count (*p* ≤ 0.009) when it was compared with the group with non-complicated appendicitis. Additionally, higher levels of neutrophils, bands, prothrombin time, INR, partial thromboplastin time values, glucose, chlorine, potassium, sodium, and magnesium were observed in complicated appendicitis (*p* < 0.05).

### 3.3. Multivariate Assessment of Data Integrated into Indices and Ratios Related to Appendicitis Outcome: Uncomplicated/Complicated and Non-Perforated/Perforated

The results of the factor analysis by stage of surgical and histopathological diagnosis were evaluated in terms of the corresponding weights per variable according to the components. The KMO is a measure of sampling adequacy used in factor analysis to determine if the data are suitable for this type of analysis. It assesses the proportion of variance among observed variables that are common, which is essential for factor analysis. Only KMO values greater than 0.6 were considered to define the best models, combinations of variables, for the two diagnostic groups. Six models were obtained for the purposes of both surgical and histopathological diagnosis (Supplementary Tables S1 and S2). Model 3 was selected as it exhibited the highest KMO value for both classifications. This model considered the following variables: prothrombin time (seconds), INR (%), partial thromboplastin time (seconds), urea (mg/dL), serum creatinine (mg/dL), neutrophils (%), lymphocytes (%), and glucose (mg/dL). The analysis yielded three components for each stage of each classification (Supplementary Tables S3 and S4).

In the Model 3, for surgical diagnosis, the stage IV demonstrated the optimal KMO with a value of 0.686 (Supplementary Table S2), which was derived from the combination of three components and exhibited an accumulated variance of 77.512% (Supplementary Table S3). In the case of histopathological diagnosis, perforated (Stage IV) demonstrated the optimal KMO value of 0.782 (Supplementary Table S1); this was the result of a combination of two components and an accumulated variance of 78.183% (Supplementary Table S3). Supplementary Table S4 presents the distribution of the components in this model with their respective weights and stratified by diagnostic classification.

### 3.4. Receiver Operating Characteristic Curve Analysis: Performance of Variables and Indices Associated with the Appendicitis Outcome

The variables with the highest association values with complicated and/or perforated appendicitis, and grouped according with the factor analysis, were integrated in indices and ratios. These data were Lymphocytes (%), Neutrophils (%), Glucose (mg/dl), and Serum creatinine (mg/dl), and they were used to construct ROC curves. On the same way, NLR, PLR, and SII were calculated and used to comparatively evaluate the performance of the suggested ratios and/or indices. The results obtained are shown in Table 3 and Table 4.

**Figure 4:**
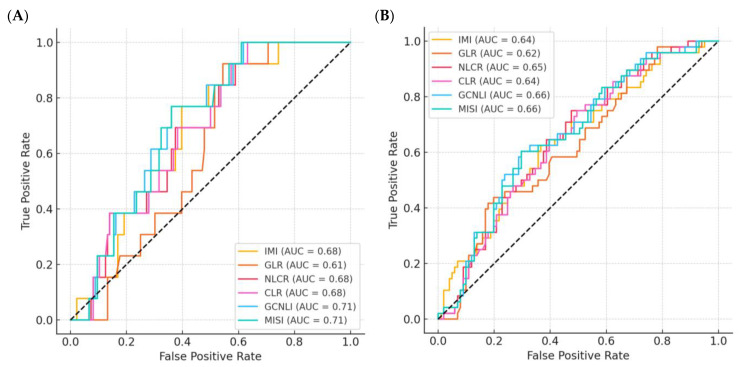
ROC curves for the diagnostic performance of all evaluated indices and ratios. (**A**) ROC curves based on histopathological diagnosis of acute appendicitis, showing the discriminatory ability of each index to distinguish between non-perforated and perforated cases. (**B**) ROC curves based on surgical diagnosis, evaluating the ability of each index to differentiate between uncomplicated and complicated appendicitis. The area under the curve (AUC) values demonstrate the comparative diagnostic accuracy of the proposed indices and ratios. IMI: inflammatory metabolic index. GLR: Glucose-Lymphocyte Ratio; NLCR: Neutrophil-Lymphocyte Creatinine Ratio; CLR: Creatinine-Lymphocyte Ratio; GCNLI: Glucose-Creatinine Neutrophil-Lymphocyte Index; MISI: Metabolic-Inflammatory Stress Index.

Considering the histopathological diagnosis, the AUC values of NLR, PLR, and SII ranged between 0.600 and 0.662. NLR had the higher AUC (AUC = 0.662; 95% CI: 0.548–0.775; *p* = 0.014). The cutoffs calculated for NLR, PLR, and SII were 5.015, 147.42, and 1463.02, respectively. The sensitivity values at the fixed cutoff values ranged between 95.24% and 100%, whereas the specificity values ranged between 26.76% and 32.26%. The results of the calculated OR using PLR showed that patients with PLR values above the fixed cutoff had an increased risk of perforated appendicitis up to nine times among the study population (OR = 9.5; 95% CI: 1.3–72.4; *p* = 0.009). The AUC values of IMI, GLR, NLCR, CLR, GCNLI, and MISI were 0.688, 0.721, 0.699, 0.720, 0.597, and 0.742, respectively (Table 4 and Figure 4A). The cutoffs calculated for these were 658.71 (IMI), 117.46 (GLR), 9.66 (NLCR), 0.64 (CLR), 905.51 (GCNLI), and 83.82 (MISI). For the suggested indices and ratios, MISI had the highest AUC (AUC = 0.742; 95% CI: 0.628–0.857; *p* ≤ 0.0005). The sensitivities at the set cutoffs ranged from 57.89% to 94.74%, with IMI having the highest value, while the specificity values ranged from 33.9% to 75.14%, with NLCR having the higher value. The OR calculated using IMI showed that patients with IMI values above the set cutoff had up to a tenfold increased risk of perforated appendicitis in the study population (OR = 10.45; 95% CI: 1.36–80.08; *p* = 0.012).

According to the surgical diagnosis, the AUC values of NLR, PLR, and SII ranged between 0.667 and 0.679 (Table 3). NLR was found to have the higher AUC (AUC = 0.679; 95% CI: 0.606–0.751; *p* ≤ 0.0001). The cutoffs calculated for NLR, PLR, and SII were 10.76, 235.35, and 3718.42, respectively. The sensitivity and specificity values for complicated appendicitis at the fixed cutoff values ranged from 33.75% to 46.34% and from 82.19% to 91.78%, respectively. The calculated OR using SII revealed that patients with SII values above the fixed cutoff had up to 5.7-fold increased odds of complicated appendicitis among the study population (OR = 5.7; 95% CI: 2.3–13.9; *p* ≤ 0.001). Concerning the surgical diagnosis classification (Table 4 and Figure 4B), the AUC values of IMI, GLR, NLCR, CLR, GCNLI, and MISI ranged from 0.632 to 0.762. GCNLI had the higher AUC (AUC = 0.762; 95% CI: 0.691–0.833; *p* ≤ 0.0001). The cutoffs calculated for these classificatory indices/ratios were 987.83, 71.74, 8.04, 0.47, 1267.7, and 54.81, respectively. The sensitivities at the established cutoffs ranged from 46.67% to 68.15%, with GCNLI having the highest value; the specificity values for IMI, GLR, NLCR, CLR, GCNLI, and MISI were 78.69%, 75.81%, 83.61%, 64.52%, 73.77%, and 72.58%, respectively. The OR calculation showed that patients with GLR values above the fixed cutoff had up to a sixfold increased odds of complicated appendicitis among the study population (OR = 6.1; 95% CI: 3.07–11.97; *p* ≤ 0.001).

Finally, a *post-hoc* power analysis was conducted to evaluate the adequacy of the sample size used in this study, particularly in relation to the main indices associated with complicated and perforated appendicitis. The results are displayed in Table 5.

All indices demonstrated high statistical power exceeding 0.93, with several reaching 1.00, indicating a very low likelihood of Type II error. For example, IMI showed a power of 1.00, and the MISI had a power of 0.978, supporting the reliability of the associations found. These results suggest that the sample size was sufficient to detect the observed effects with a high degree of confidence. In addition, the adequacy of the sample for the factor analysis was evaluated using the Kaiser-Meyer-Olkin (KMO) measure of sampling adequacy. All models considered for the development of the new indices exhibited KMO values above 0.6, which is generally considered acceptable for factor analysis. Moreover, with a total sample of 246 patients and fewer than ten variables included per model, the subject-to-variable ratio met the commonly accepted criteria for stable and interpretable factor solutions. These considerations support the robustness and validity of the multivariate models and the indices derived from them.

## 4. Discussion

Acute appendicitis is the most common abdominal surgical emergency worldwide [2]. Prompt and precise diagnosis is crucial, as it can significantly decrease the risk of perforation and other serious complications associated with this condition [1]. The pathophysiology of appendicitis involves important biochemical parameters that have not been taken into consideration in previous scales. Since there is currently no specific biomarker available for diagnosing and/or predicting acute appendicitis, this study aimed to evaluate the basic laboratory tests of patients with acute appendicitis and integrate biochemical variables into their diagnosis. The diagnostic/prognosis values of these variables were also compared with existing indices.

The analysis of the general characteristics of the studied population made it possible to identify some noteworthy points: the lifetime risk of experiencing acute appendicitis in our study was slightly higher in men compared to women (51.6% vs. 48.4%), which differs from previous studies (12% vs. 23.1%) [6,10]. Men are also more prone to perforated appendicitis than women [2,11], which aligns to some extent with our study’s outcomes. Acute appendicitis generally affects individuals aged 10–19 years [10], but our results differed slightly, revealing a higher incidence among adults over 19 years, with a mean age of 25 years. Although prior research suggests that the LA should be favored over the open approach [37,38,39] and is considered the gold standard treatment [39], only 3.25% of the surgical procedures in this study were performed using LA. It is important to note that the hospital where the study was conducted is a public second-level facility located in central-northern Mexico and operates under the jurisdiction of the Health Secretariat. The hospital does not possess laparoscopic equipment, and the patient population it serves typically lacks social security and has limited economic resources. While patients are offered the option of an LA, this requires the rental of equipment, the cost of which must be covered by the patient. As a result, open appendectomy is favored, particularly in cases of complicated appendicitis. This scenario reflects a common reality in many hospitals in low- and middle-income countries, where resource limitations significantly influence surgical decision-making.

In this study, the univariate analysis showed consistent differences in variables such as percentage of lymphocytes, prothrombin time, INR, partial thromboplastin time, and glucose levels between groups of perforated/non-perforated appendicitis and complicated/non-complicated appendicitis. Other variables, such as leukocytes, neutrophils, bands, lymphocyte counts, serum creatinine, and some electrolytes (chloride, potassium, sodium, and magnesium), remained significant only in one classification of acute appendicitis. These findings highlight the distinct profiles of laboratory markers in different classifications of acute appendicitis, highlighting the importance of comprehensive diagnostic criteria to accurately distinguish between perforated and non-perforated as well as complicated and non-complicated cases.

In our results, in the multivariate assessment Model 3, identified as the best model in this study, grouped significant variables into three primary components used to guide the construction of the proposed indices: Component One, which included prothrombin time, INR, and partial thromboplastin time; Component Two, with urea, serum creatinine, and glucose; and Component Three, which grouped neutrophils and lymphocytes. These three components provide important insights into acute appendicitis diagnosis. Component One is particularly significant, as it explains a significant proportion of the etiology, with prothrombin time, INR, and partial thromboplastin time reflecting the endothelial dysfunction that triggers the inflammatory response and activates coagulation, platelets, and inflammatory cells [40]. This phenomenon has been thoroughly studied in sepsis but is less explored in acute appendicitis [41]. Guana et al. associated prolonged prothrombin time with complicated appendicitis, possibly due to impaired vitamin K absorption resulting from enteric inflammation [42]. Unfortunately, in our study, the lack of information, like the presence of liver disease, recent blood transfusions, and nonsteroidal anti-inflammatory drugs use, limited our ability to use coagulation times as variables within the suggested indices. Component Two consistently identified urea, serum creatinine, and glucose as the second-most important group of variables, and Component Three, with neutrophils and lymphocytes. In this sense, a previous report carried out by Marzuillo et al. found that 7.4% of pediatric patients with acute appendicitis developed mild prerenal acute kidney injury (elevated creatinine serum levels), linked to vomiting, dehydration above 5%, fever over 38.5 °C, and elevated C-reactive protein and neutrophil levels [43] which may to explain the importance of serum creatinine levels observed in our study. The grouping of these variables through PCA provided a statistically grounded rationale for index construction. Variables within the same component demonstrated shared variance and clinical coherence, allowing their combination into unified scores. For example, IMI multiplies glucose and neutrophil percentages, two variables that clustered strongly within the same component, emphasizing their synergistic role in the metabolic-inflammatory response observed in complicated appendicitis. Similarly, MISI integrates variables from distinct components related to immune and metabolic activation. This approach reduced dimensionality while preserving biological interpretability. The variables in Components Two and Three also highlight the immune response to acute appendicitis and stress-induced hyperglycemia from HPA [32,33,34]. This last issue is not unexpected, but it has not been explored in the appendicitis diagnosis context. It is well accepted that acute appendicitis can trigger stress, activating the HPA axis and increasing the secretion of hormones like cortisol, norepinephrine, and glucagon [14,32]. This response boosts hepatic gluconeogenesis and reduces insulin sensitivity, resulting in hyperglycemia [32,33], which agrees with our results. Altogether, the contribution of these components enabled the construction of six indices and ratios (IMI, GLR, NLCR, CLR, GCNLI, and MISI), translating the multivariate structure into tools with potential clinical applicability, as discussed in the following sections.

The results of ROC analysis in our study showed that the AUC for IMI, GLR, NLCR, CLR, and MISI were higher than those for NLR, PLR, and SII for the classification of non-perforated and perforated appendicitis according to the established cut-off points (Table 4 and Table 5). Similarly, the relationship between sensitivity, specificity, NPV, and PPV values, based on the set cutoff points, of the suggested indices and ratio was more consistent than that shown for NLR, PLR, and SII for this group. Even the lowest specificity value based on the defined cut-off points of IMI, GLR, NLCR, CLR, and MISI was higher than those found in the existing indices and ratios (NLR, PLR, and SII). The findings of the OR calculated using GLR indicate that patients with GLR values above the specified cutoff points had up to a sixfold increased risk of perforation in the study sample. This demonstrates that the suggested indices and ratios have greater predictive power for perforated appendicitis. In the same way, the results for the classification of surgical diagnosis (complicated and non-complicated) based on the fixed cutoff value, demonstrated that IMI, GLR, and GCNLI have higher AUC values and greater predictive power for complicated appendicitis (Table 4). Undoubtedly, the relationship between sensitivity, specificity, NPV, and PPV values on the set threshold values in the suggested indices and radius was found to be more steadfast than that shown for NLR, PLR, and SII for this classification (Table 3).

Although the present study exclusively included patients with confirmed acute appendicitis to evaluate biochemical indices associated with disease severity, it is important to acknowledge that, in clinical practice, several conditions may mimic appendicitis, particularly during initial evaluation. Differential diagnoses include mesenteric lymphadenitis, Meckel’s diverticulitis, Crohn’s disease, pelvic inflammatory disease, ovarian torsion, and urinary tract infections. While these entities were beyond the scope of our analysis, their clinical overlap highlights the importance of accurate triage tools prior to applying stratification indices such as that developed in this work.

Finally, an important advantage of the proposed indices and ratios is their practicality in emergency settings. All required parameters, such as blood glucose, leukocyte count, neutrophils, lymphocytes, and creatinine, are part of the routine laboratory tests typically ordered during the initial evaluation of patients with suspected acute appendicitis. As such, these indices can be calculated quickly, without the need for specialized tests or additional resources, making them highly suitable for real-time risk stratification and decision-making in resource-limited or high-demand emergency departments.

### 4.1. Future Directions

A potential avenue for enhancing the clinical utility of the proposed indices and ratios is their integration into electronic medical record (EMR) systems. Embedding automated calculations of IMI and MISI into EMRs could streamline diagnostic workflows and support real-time decision-making, particularly in emergency settings where rapid assessment is critical. Calculators such as the proposed in this work (https://calculator.labmedmol.mx/, accessed on 15 July 2025) may facilitate this integration, and it may be especially valuable in resource-limited hospitals, where access to advanced imaging or specialist consultation may be constrained. By leveraging routinely available laboratory data, these indices could serve as early warning tools, prompting clinicians to prioritize high-risk patients and optimize care pathways. Future work should explore the feasibility of such integration and its impact on diagnostic accuracy, time to intervention, and patient outcomes.

In addition, we are currently designing a prospective, multicenter validation protocol to assess the real-time performance of IMI and MISI in diverse healthcare settings. This planned study will also evaluate threshold adjustments across populations and explore the potential for integrating these indices with existing clinical scoring systems. These next steps aim to support the external validation and generalizability of our findings and to move toward the clinical implementation of these indices.

### 4.2. Study Limitations

It is important to note that our study exclusively included patients with a confirmed diagnosis of acute appendicitis, with the primary aim of developing laboratory-based indices and ratios to assess disease severity, specifically the risk of complication or perforation. Therefore, traditional diagnostic scores such as the Alvarado or AIR scale, which are designed to aid in the initial identification of appendicitis among patients presenting with undifferentiated abdominal pain, were not used as comparators. Our indices are intended to complement clinical decision-making after the diagnostic threshold has been crossed, rather than to replace or rival diagnostic scoring systems. Additionally, we acknowledge that IMI and MISI may be influenced by extrinsic factors such as stress-related hyperglycemia or underlying conditions like diabetes mellitus, which could affect glucose levels independently of appendiceal pathology. These limitations must be considered in the interpretation and future application of our findings, particularly in settings with a high prevalence of metabolic comorbidities.

Advanced inflammatory biomarkers such as procalcitonin, interleukin-6 (IL-6), and butyrylcholinesterase have been proposed in some studies for identifying severe infections or predicting surgical complications [44,45,46]. However, their diagnostic utility in acute appendicitis remains limited and inconsistent, and they are not routinely used in clinical practice. Moreover, due to cost constraints and availability issues, these markers are not measured in our hospital setting. According to the above, our study focused on widely available laboratory parameters to ensure clinical applicability. Future research may explore the complementary role of these biomarkers in resource-equipped centers, particularly for prognostic purposes.

Furthermore, as the study was conducted at a single center in central-northern Mexico, the patient cohort may reflect regional or population-specific trends in inflammatory and metabolic profiles. Factors such as nutritional status, prevalence of metabolic conditions, and access to early surgical care can influence baseline laboratory values. These differences may affect the generalizability of the proposed indices in other settings. Future validation efforts should consider potential population-level variability and explore necessary adjustments to index thresholds where appropriate. Finally, the retrospective design of this study imposes inherent limitations regarding causal inference. While the statistical associations are robust, prospective validation in diverse clinical environments remains necessary to confirm the predictive value of IMI and MISI and their potential utility in improving patient outcomes.

## 5. Conclusions

Our findings identified key biochemical markers like urea, serum creatinine, glucose, neutrophils, and lymphocytes that help distinguish between perforated/non-perforated and complicated/non-complicated cases. The newly developed indices, such as IMI and MISI, which include inflammatory and metabolic parameters, demonstrated to be effective biomarkers for complicated and perforated appendicitis. IMI provides predictive capability, while MISI offers specificity and significant AUC values for both histopathological and surgical diagnoses, therefore providing a more accurate tool for the classification of appendicitis. The communality identified through factor analysis may lead to the construction of new classification indexes for this disease. Given that our study was conducted at a single center, we acknowledge the need for external validation of the proposed indices and ratios and validation of them as markers of acute appendicitis.

## Figures and Tables

**Table 1:** Calculation of metabolic and inflammatory indices included in the study.

Name Indices/Ratios	Variables Required for the Calculation	Equation
IMI Inflammatory Metabolic Index	Glucose level Neutrophil count Total Lymphocyte count	$\frac{\left(Glucose\;level)\;\times \;(Neutrophil\;count\right)}{Total\;Lymphocyte\;count}$
GLR Glucose-Lymphocyte Ratio	Glucose level Lymphocyte count	$\frac{Glucose\;level}{Lymphocyte\;count}$
NLCR Neutrophil-Lymphocyte Creatinine Ratio	Creatinine level Neutrophil count Total Lymphocyte count	$\frac{\left(Creatinine\;level)\;\times \;(Neutrophil\;count\right)}{Total\;Lymphocyte\;count}$
CLR Creatinine-Lymphocyte Ratio	Creatinine level Total lymphocyte count	$\frac{Creatinine\;level}{Total\;lymphocyte\;count}$
GCNLI Glucose-Creatinine Neutrophil-Lymphocyte Index	Creatinine level Glucose level Neutrophil count Total Lymphocyte count	$\left(\frac{Glucose\;level}{Creatinine\;level})\;\times \;(\frac{Neutrophil\;count}{Total\;Lymphocyte\;count}\right)$
MISI Metabolic-Inflammatory Stress Index	Glucose level Creatinine level Total lymphocyte count	$\frac{\left(Glucose\;level)\;\times \;(Creatinine\;level\right)}{Total\;lymphocyte\;count}$

For an easy calculation of these new indices and ratios, an electronic calculator for each index and ratio is available on https://calculator.labmedmol.mx/ (accessed on 15 July 2025).

**Table 2:** General characteristics of the population.

Parameter	Histopathological Diagnosis			Surgical Diagnosis		
	Perforated Appendicitis (*n* = 21)	Non-Perforated Appendicitis (*n* = 225)	*p*-Value	Complicated Appendicitis (*n* = 76)	Non-Complicated Appendicitis (*n* = 170)	*p*-Value
Sex						
Male, *n* (%)	11 (4.47)	116 (47.16)	0.199	36 (14.63)	91 (37)	0.199
Female, *n* (%)	10 (4.06)	109 (44.31)		40 (16.26)	79 (32.11)	
Age (years)	36.67 ± 24.58	23.68 ± 18.43	0.011 *	24.75 ± 20.23	24.90 ± 17.23	0.337
Hospitalization (days)	4.71 ± 3.64	3.10 ± 2.75	0.079	3.46 ± 2.74	2.45 ± 2.96	<0.001 *
Erythrocyte (106/μL)	4.78 ± 0.52	4.82 ± 0.72	0.53	4.77 ± 0.64	4.92 ± 0.83	0.06
Hemoglobin (g/dL)	14.18 ± 162	14.07 ± 2.14	0.91	13.87 ± 14.53	14.53 ± 1.97	0.02
Hematocrit (%)	40.73 ± 4.35	40.81 ± 5.05	0.95	10.41 ± 4.97	41.68 ± 4.94	0.07
Mean corpuscular volume (fL)	85.25 ± 3.24	85.69 ± 25.64	0.44	86.45 ± 29.2	83.86 ± 5.56	0.86
Mean corpuscular hemoglobin (pg/cell)	29.66 ± 1.15	29.02 ± 2.32	0.16	28.99 ± 2.09	29.25 ± 2.58	0.16
Mean corpuscular hemoglobin concentration (g/dL)	34.81 ± 1.3	34.58 ± 1.37	0.4	34.51 ± 1.3	34.82 ± 1.49	0.03
Red cell distribution width (%)	12.87 ± 0.93	14.32 ± 20.45	0.64	14.84 ± 23.84	12.73 ± 1.8	0.02
Platelets (10^3^/μL)	245.52 ± 81.02	273.91 ± 80.15	0.12	274.15 ± 84.18	265.43 ± 71.5	0.75
Leukocytes (10^3^/μL)	13.34 ± 4.63	14.77 ± 5.10	0.3	15.26 ± 5.3	13.25 ± 4.21	0.008 *
Lymphocytes (%)	0.1 ± 0.57	1.52 ± 0.80	<0.001 *	1.35 ± 0.79	1.77 ± 0.75	<0.001 *
Neutrophils (%)	11.55 ± 4.21	11.85 ± 4.97	0.86	12.45 ± 5.12	10.43 ± 4.09	0.001 *
Leukocyte differential						
Bands (10^3^/μL)	8 ± 9.5	7.45 ± 10.1	0.75	8.64 ± 11.21	4.71 ± 5.49	0.02 *
Segmented (10^3^/μL)	8 ± 79.07	75.66 ± 12.29	0.23	76.51 ± 12.06	74.51 ± 12.79	0.32
Monocyte (10^3^/μL)	4.54 ± 2.66	4.79 ± 3.19	0.8	4.73 ± 2.96	4.85 ± 3.59	0.9
Lymphocytes (10^3^/μL)	9.64 ± 5.2	12.8 ± 9.17	0.29	11.34 ± 7.98	15.54 ± 10.47	0.009 *
Prothrombin time (seconds)	15.62 ± 2.23	17.57 ± 5.47	0.02 *	16.22 ± 2.95	14.89 ± 1.84	<0.001 *
International normalized ratio (%)	1.9 ± 0.47	1.21 ± 0.2	0.018 *	1.27 ± 0.26	1.15 ± 0.16	<0.001 *
Partial thromboplastin time (seconds)	17.57 ± 5.5	33.45 ± 5.53	0.008 *	34.74 ± 6.17	31.82 ± 3.84	<0.001 *
Glucose (mg/dL)	125.73 ± 30.49	109.27 ± 39.66	0.001 *	117.01 ± 43.58	96.65 ± 20.61	<0.001 *
Urea (mg/dL)	40 ± 46.48	26.82 ± 17.66	0.52	28.63 ± 23.28	26.69 ± 19.25	0.46
Serum creatinine (mg/dL)	0.96 ± 0.41	0.77 ± 0.57	0.003 *	0.77 ± 0.46	0.84 ± 0.74	0.95
Phosphorous (mg/dL)	3.47 ± 1.74	4.53 ± 9.28	0.24	4.68 ± 10.51	3.86 ± 2.31	0.76
Calcium (mg/dL)	14.54 ± 21.8	9.11 ± 1.29	0.25	9.8 ± 8.42	9.35 ± 0.61	0.21
Chlorine (mmol/L)	98.78 ± 6.16	101.3 ± 9.37	0.059	100.23 ± 10.56	102.95 ± 3.9	0.014 *
Potassium (mmol/L)	3.82 ± 0.55	3.9 ± 0.43	0.22	3.94 ± 0.48	3.77 ± 0.33	0.018 *
Sodium (mmol/L)	133.84 ± 5.33	135.41 ± 10.98	0.09	135.37 ± 5.44	135.04 ± 17.3	0.035 *
Magnesium (mg/dL)	2.08 ± 0.37	1.98 ± 0.4	0.57	2.01 ± 0.33	1.97 ± 0.52	0.043 *
Blood urea nitrogen (mg/dL)	18.66 ± 21.77	12.49 ± 7.48	0.5	13.59 ± 11.17	11.94 ± 5.93	0.51

Significant *p*-values are highlighted with an asterisk.

**Table 3:** The use of biomarkers in predicting complicated and perforated appendicitis.

Parameter	Histopathological Diagnosis			Surgical Diagnosis		
	NLR	PLR	SII	NLR	PLR	SII
Area under ROC curve	0.662	0.648	0.600	0.679	0.677	0.667
Cutoff	5.015	147.42	1463.02	10.76	235.355	3718.42
*p*-value	0.014 *	0.025 *	0.1300	<0.000 *	<0.000 *	<0.000 *
Sensitivity	95.45	95.24	100	45.96	46.34	33.75
Specificity	26.76	32.26	31.13	82.19	83.78	91.78
PPV	11.86	11.98	12.57	85.06	86.36	90
NPV	98.28	98.59	100	40.82	41.33	38.73
Youden index	22.21	27.5	31.13	28.15	30.12	25.53
Odds ratio (95% CI)	7.7 (1.0–58.4)	9.5 (1.3–72.4)	+inf	3.9 (1.99–7.7)	4.5 (2.2–8.0)	5.7 (2.3–13.9)
*p*-value	0.006 *	0.009 *	0.006 *	<0.001 *	<0.001 *	<0.001 *

NLR: neutrophil lymphocyte ratio; PLR: platelet lymphocyte ratio; SII: systemic immune inflammation index; PPV: positive predictive value; NPV: negative predictive value. Significant *p*-values are highlighted with an asterisk.

**Table 4:** Suggested biomarkers for complicated and perforated appendicitis.

Parameter	Histopathological Diagnosis						Surgical Diagnosis					
	IMI	GLR	NLCR	CLR	GCNLI	MISI	IMI	GLR	NLCR	CLR	GCNLI	MISI
Area under ROC curve	0.688	0.721	0.699	0.720	0.597	0.742	0.753	0.743	0.677	0.632	0.762	0.676
*p*-value	0.007 *	0.002 *	0.004 *	0.0016 *	0.17	<0.0005 *	<0.0001 *	<0.0001 *	<0.0001 *	0.003 *	<0.0001 *	<0.0001 *
Cutoff	658.71	117.46	9.66	0.64	905.51	83.82	987.83	71.74	8.04	0.47	1267.7	54.81
Sensitivity	94.74	68.42	57.89	73.68	89.47	68.42	58.52	65.94	46.67	64.49	68.15	60.15
Specificity	36.72	73.48	75.14	62.98	33.9	72.38	78.69	75.81	83.61	64.52	73.77	72.58
PPV	13.84	21.31	20	17.28	12.69	20.63	85.87	85.85	86.3	80.18	85.18	83
NPV	98.48	95.68	94.3	95.8	96.77	95.62	46.15	50	41.46	44.94	51.14	45
Youden index	31.46	41.9	33.04	36.67	23.37	40.8	37.21	41.75	30.27	29.01	41.92	32.73
Odds ratio (95% CI)	10.45 (1.36–80.08)	6.01 (2.16–16.68)	4.2 (1.57–10.99)	4.76 (1.64–13.82)	4.36 (0.97–19.5)	5.68 (2.04–15.75)	5.21 (2.58–10.51)	6.1 (3.07– 11.97)	4.46 (2.09–9.52)	3.3 (1.76–6.18)	6.02 (3.06–11.83)	3.99 (2.08–7.68)
*p*-value	0.012 *	<0.001 *	0.005 *	0.004 *	0.068	<0.001 *	<0.001 *	<0.001 *	<0.001 *	<0.001 *	<0.001 *	<0.001 *

IMI: inflammatory metabolic index. GLR: Glucose-Lymphocyte Ratio; NLCR: Neutrophil-Lymphocyte Creatinine Ratio; CLR: Creatinine-Lymphocyte Ratio; GCNLI: Glucose-Creatinine Neutrophil-Lymphocyte Index; MISI: Metabolic-Inflammatory Stress Index; PPV: positive predictive value; NPV: negative predictive value. Significant *p*-values are highlighted with an asterisk.

**Table 5:** Statistical power calculations for the proposed indices.

Index	Odds Ratio	Group 1 (*n*)	Group 2 (*n*)	Effect Size Formula	Power Formula	Power Formula (Rounded)
Histopathological						
IMI	10.45	21	225	1.29375	0.99990	1.000
GLR	6.01	21	225	0.98877	0.99119	0.991
NLCR	4.2	21	225	0.79120	0.93416	0.934
CLR	4.76	21	225	0.86021	0.96485	0.965
GCNLI	4.36	21	225	0.81182	0.94497	0.945
MISI	5.68	21	225	0.95763	0.98735	0.987
Surgical						
IMI	5.21	76	170	0.91001	1.00000	1.000
GLR	6.1	76	170	0.99696	1.00000	1.000
NLCR	4.46	76	170	0.82432	0.99997	1.000
CLR	3.3	76	170	0.65824	0.99753	0.998
GCNLI	6.02	76	170	0.98968	1.00000	1.000
MISI	3.99	76	170	0.76292	0.99982	1.000

IMI: inflammatory metabolic index. GLR: Glucose-Lymphocyte Ratio; NLCR: Neutrophil-Lymphocyte Creatinine Ratio; CLR: Creatinine-Lymphocyte Ratio; GCNLI: Glucose-Creatinine Neutrophil-Lymphocyte Index; MISI: Metabolic-Inflammatory Stress Index.

## Data Availability

The original contributions presented in this study are included in the article/Supplementary Materials. Further inquiries can be directed to the corresponding author.

## Supplementary Materials

The following supporting information can be downloaded at: https://www.mdpi.com/article/10.3390/jcm14155281/s1, Table S1: Combinations of variables analyzed (Models) through factor analysis for histopathological diagnosis, and their KMO values; Table S2: Combinations of variables analyzed (Models) through factor analysis, for surgical diagnosis, and their KMO values; Table S3: Principal component analysis of significant variables associated with histopathological and surgical diagnosis of appendicitis. Initial autovalues (total, variance and accumulated variance) for the Model 3 are shown by appendicitis stage and classified according to the histopathological and surgical diagnosis; Table S4: Rotated Component Matrix (Varimax) for variables associated with histopathological and surgical diagnosis of acute appendicitis: Model 3.

## Abbreviations

The following abbreviations are used in this manuscript:

IMI	Inflammatory Metabolic Index
GLR	Glucose-Lymphocyte Ratio
NLCR	Neutrophil-Lymphocyte Creatinine Ratio
CLR	Creatinine-Lymphocyte Ratio
GCNLI	Glucose-Creatinine Neutrophil-Lymphocyte Index
MISI	Metabolic-Inflammatory Stress Index
NLR	Neutrophil lymphocyte ratio
PLR	Platelet lymphocyte ratio
SII	Systemic immune inflammation index

## References

[1] Snyder M.J., Guthrie M., Cagle S. (2018). Acute Appendicitis: Efficient Diagnosis and Management. Am. Fam. Physician.

[2] Moris D., Paulson E.K., Pappas T.N. (2021). Diagnosis and Management of Acute Appendicitis in Adults: A Review. JAMA.

[3] IMSS COM. 561 Identificar Síntomas de Apendicitis y Acudir de Manera Oportuna al Servicio de Urgencias, Favorece Atención Médica: IMSS. https://www.gob.mx/imss/prensa/com-561-identificar-sintomas-de-apendicitis-y-acudir-de-manera-oportuna-al-servicio-de-urgencias-favorece-atencion-medica-imss#:~:text=La%20incidencia%20de%20padecer%20apendicitis%20aguda%20es%201%20por%20cada%201000%20personas.

[4] Pogorelic Z., Cohadzic T. (2023). A Bizarre Cause of Acute Appendicitis in a Pediatric Patient: An Ingested Tooth. Children.

[5] Sousa J., Hawkins R., Shenoy A., Petroze R., Mustafa M., Taylor J., Larson S., Islam S. (2022). Enterobius vermicularis-associated appendicitis: A 22-year case series and comprehensive review of the literature. J. Pediatr. Surg..

[6] Teoule P., Laffolie J., Rolle U., Reissfelder C. (2020). Acute Appendicitis in Childhood and Adulthood. Dtsch. Arztebl. Int..

[7] Soybel D., Norton J.A. (2003). Appendix. Surgery: Basic Science and Clinical Evidence.

[8] Buschard K., Kjaeldgaard A. (1973). Investigation and analysis of the position, fixation, length and embryology of the vermiform appendix. Acta Chir. Scand..

[9] Livingston E.H., Woodward W.A., Sarosi G.A., Haley R.W. (2007). Disconnect between incidence of nonperforated and perforated appendicitis: Implications for pathophysiology and management. Ann. Surg..

[10] Addiss D.G., Shaffer N., Fowler B.S., Tauxe R.V. (1990). The epidemiology of appendicitis and appendectomy in the United States. Am. J. Epidemiol..

[11] Golz R.A., Flum D.R., Sanchez S.E., Liu X., Donovan C., Drake F.T. (2020). Geographic Association Between Incidence of Acute Appendicitis and Socioeconomic Status. JAMA Surg..

[12] Ohle R., O’Reilly F., O’Brien K.K., Fahey T., Dimitrov B.D. (2011). The Alvarado score for predicting acute appendicitis: A systematic review. BMC Med..

[13] Markar S.R., Vidal-Diez A., Patel K., Maynard W., Tukanova K., Murray A., Holt P.J., Karthikesalingam A., Hanna G.B. (2019). Comparison of Surgical Intervention and Mortality for Seven Surgical Emergencies in England and the United States. Ann. Surg..

[14] Harris J., Fleming C.A., Stassen P.N., Mullen D., Mohan H., Foley J., Heeney A., Nugent E., Schmidt K., Mealy K. (2022). A comparison of intra-operative diagnosis to histopathological diagnosis of acute appendicitis in paediatric and adult cohorts: An analysis of over 1000 patients. Ir. J. Med. Sci..

[15] Al-Gaithy Z.K. (2012). Clinical value of total white blood cells and neutrophil counts in patients with suspected appendicitis: Retrospective study. World J. Emerg. Surg..

[16] Jung S.K., Rhee D.Y., Lee W.J., Woo S.H., Seol S.H., Kim D.H., Choi S.P. (2017). Neutrophil-to-lymphocyte count ratio is associated with perforated appendicitis in elderly patients of emergency department. Aging Clin. Exp. Res..

[17] Valga F., Monzon T., Henriquez F., Santana-Del-Pino A., Anton-Perez G. (2020). Platelet-to-lymphocyte and neutrophil-to-lymphocyte ratios as markers of erythropoietin resistance in chronic haemodialysis patients: A multicentre cross-sectional study. Nefrologia (Engl. Ed.).

[18] Atum M., Alagoz G. (2020). Neutrophil-to-lymphocyte Ratio and Platelet-to-lymphocyte Ratio in Patients with Retinal Artery Occlusion. J. Ophthalmic Vis. Res..

[19] Aydogdu B., Azizoglu M., Arslan S., Aydogdu G., Basuguy E., Salik F., Okten M., Hanifi-Okur M. (2023). A novel diagnostic scoring system for pediatric appendicitis based on age and sex-adjusted hematological parameters. Gac. Med. Mex..

[20] Tekeli A., Caliskan M.B., Bahadir G.B., Erdemir O.K. (2023). Evaluation of systemic immune-inflammation index efficacy in predicting complicated appendicitis in pediatric emergency department. Ulus. Travma Acil Cerrahi Derg..

[21] Zarog M., O’Leary P., Kiernan M., Bolger J., Tibbitts P., Coffey S., Byrnes G., Peirce C., Dunne C., Coffey C. (2023). Circulating fibrocyte percentage and neutrophil-lymphocyte ratio are accurate biomarkers of uncomplicated and complicated appendicitis: A prospective cohort study. Int. J. Surg..

[22] Zviedre A., Engelis A., Tretjakovs P., Jurka A., Zile I., Petersons A. (2016). Role of serum cytokines in acute appendicitis and acute mesenteric lymphadenitis among children. Medicina.

[23] Pehlivanli F., Aydin O. (2019). Role of Platelet to Lymphocyte Ratio as a Biomedical Marker for the Pre-Operative Diagnosis of Acute Appendicitis. Surg. Infect..

[24] Celik B., Nalcacioglu H., Ozcatal M., Altuner Torun Y. (2019). Role of neutrophil-to-lymphocyte ratio and platelet-to-lymphocyte ratio in identifying complicated appendicitis in the pediatric emergency department. Ulus. Travma Acil Cerrahi Derg..

[25] Gil-Vargas M., Cruz-Pena I., Saavedra-Pacheco M.S. (2022). Sensibilidad y especificidad del indice neutrofilo/linfocito en pacientes pediatricos con apendicitis aguda complicada. Cirugía Y Cir..

[26] Seclén-Hidalgo D., Perales-Che-León F.A., Díaz-Vélez C. (2018). Valor diagnóstico de la razón neutrófilos-linfoctios para identificar apendicitis aguda complicada. Rev. Cuerpo Med. HNAAA.

[27] Guneylioglu M.M., Gungor A., Goktug A., Uner C., Bodur I., Yaradilmis R.M., Ozturk B., Sen Z.S., Tuygun N. (2022). Evaluation of the efficiency of the systemic immune-inflammation index in differentiating parapneumonic effusion from empyema. Pediatr. Pulmonol..

[28] Wang R.H., Wen W.X., Jiang Z.P., Du Z.P., Ma Z.H., Lu A.L., Li H.P., Yuan F., Wu S.B., Guo J.W. (2023). The clinical value of neutrophil-to-lymphocyte ratio (NLR), systemic immune-inflammation index (SII), platelet-to-lymphocyte ratio (PLR) and systemic inflammation response index (SIRI) for predicting the occurrence and severity of pneumonia in patients with intracerebral hemorrhage. Front. Immunol..

[29] Alvarado A. (1986). A practical score for the early diagnosis of acute appendicitis. Ann. Emerg. Med..

[30] Samuel M. (2002). Pediatric appendicitis score. J. Pediatr. Surg..

[31] Gorter R.R., Eker H.H., Gorter-Stam M.A., Abis G.S., Acharya A., Ankersmit M., Antoniou S.A., Arolfo S., Babic B., Boni L. (2016). Diagnosis and management of acute appendicitis. EAES consensus development conference 2015. Surg. Endosc..

[32] Marik P.E., Raghavan M. (2004). Stress-hyperglycemia, insulin and immunomodulation in sepsis. Intensive Care Med..

[33] Hall J.E. (2016). Guyton and Hall Textbook of Medical Physiology.

[34] Silverman M.N., Pearce B.D., Biron C.A., Miller A.H. (2005). Immune modulation of the hypothalamic-pituitary-adrenal (HPA) axis during viral infection. Viral Immunol..

[35] John G.R. (2017). Rosai and Ackerman’s Surgical Pathology.

[36] Carr N.J. (2000). The pathology of acute appendicitis. Ann. Diagn. Pathol..

[37] Aziz O., Athanasiou T., Tekkis P.P., Purkayastha S., Haddow J., Malinovski V., Paraskeva P., Darzi A. (2006). Laparoscopic versus open appendectomy in children: A meta-analysis. Ann. Surg..

[38] Dai L., Shuai J. (2017). Laparoscopic versus open appendectomy in adults and children: A meta-analysis of randomized controlled trials. United Eur. Gastroenterol. J..

[39] Di Saverio S., Podda M., De Simone B., Ceresoli M., Augustin G., Gori A., Boermeester M., Sartelli M., Coccolini F., Tarasconi A. (2020). Diagnosis and treatment of acute appendicitis: 2020 update of the WSES Jerusalem guidelines. World J. Emerg. Surg..

[40] Iba T., Levy J.H. (2018). Inflammation and thrombosis: Roles of neutrophils, platelets and endothelial cells and their interactions in thrombus formation during sepsis. J. Thromb. Haemost..

[41] Iba T., Levy J.H. (2020). Sepsis-induced Coagulopathy and Disseminated Intravascular Coagulation. Anesthesiology.

[42] Guana R., Giuliano C., Pollio B., Perin S., Zambaiti E., Cerrina A., Pane A., Scottoni F., Lonati L., Garofalo S. (2023). Role of coagulation tests in the management of acute appendicitis in children. Minerva Pediatr..

[43] Marzuillo P., Coppola C., Caiazzo R., Macchini G., Di Sessa A., Guarino S., Esposito F., Miraglia Del Giudice E., Tipo V. (2022). Acute Kidney Injury in Children with Acute Appendicitis. Children.

[44] Vaziri M., Ehsanipour F., Pazouki A., Tamannaie Z., Taghavi R., Pishgahroudsari M., Jesmi F., Chaichian S. (2014). Evaluation of procalcitonin as a biomarker of diagnosis, severity and postoperative complications in adult patients with acute appendicitis. Med. J. Islam. Repub. Iran..

[45] Haghi A.R., Kasraianfard A., Monsef A., Kazemi A.S., Rahimi S., Javadi S.M.R. (2019). The diagnostic values of procalcitonin and interleukin 6 in acute appendicitis. Turk. J. Surg..

[46] Cui W., Liu H., Ni H., Qin X., Zhu L. (2019). Diagnostic accuracy of procalcitonin for overall and complicated acute appendicitis in children: A meta-analysis. Ital. J. Pediatr..

