# Supplementary material for: Inflammatory Metabolic Index and Metabolic-Inflammatory Stress Index as New Biomarkers for Complicated and Perforated Acute Appendicitis
Source: J Clin Med. 2025 Jul 25;14(15):5281. doi: 10.3390/jcm14155281 (PMC12347975; doi:10.3390/jcm14155281)
Supplement: Supplementary file 1 [file jcm-14-05281-s001.zip › 0-Supplementary Table S1.pdf]

Supplementary Table S1. Combinations of variables analyzed (Models) through factor analysis for histo-pathological diagnosis, and their KMO values.

Model/Variables	KMO value			
	**Edematous**	**Suppurative**	**Gangrenous**	**Perforated**
**Model 1**				
Platelets (10³/μL)<br>Leukocytes (10³/μL)<br>Lymphocytes (%)<br>Neutrophils (%)<br>Neutrophil-lymphocyte ratio<br>Platelet-lymphocyte ratio<br>Bands (10³/μL)<br>Prothrombin time (seconds)<br>International normalized index (%)<br>Partial thromboplastin time (seconds)<br>Glucose (mg/dl)<br>Urea (mg/dL)<br>Serum creatinine (mg/dl)<br>Sex<br>Age (years)	0.529	0.502	0.16	NT
**Model 2**				
Lymphocytes (%)<br>Neutrophils<br>Glucose (mg/dl)<br>Serum creatinine (mg/dl)	0.496*	0.505*	0.556	0.611
**Model 3**				
Prothrombin time (seconds)<br>International normalized index (%)<br>Partial thrombloplastin time (seconds)<br>Urea (mg/dL)<br>Serum creatinine (mg/dL)<br>Neutrophils (%)<br>Lymphocytes (%)<br>Glucose (mg/dL)	0.405	0.610	0.524	0.782
**Model 4**				
Neutrophils (%)<br>Glucose (mg/dl)<br>Serum creatinine (mg/dl)<br>Prothrombin time (seconds)<br>Partial thromboplastin time (seconds)<br>International normalized index (%)<br>Potassium (mmol/L)<br>Chlorine (mmol/L)<br>Sodium (mmol/L)<br>Magnesium (mg/dL)<br>Leukocytes (10³/μL)<br>Age (years)	0.393	0.536	0.355	NT
**Model 5**				
Neutrophils (%)<br>Glucose (mg/dl)<br>Serum creatinine (mg/dl)<br>Leukocytes<br>Age (years)<br>Platelets (10³/μL)	0.532	0.558	0.506	0.507
**Model 6**				
Neutrophils (%)<br>Glucose (mg/dl)<br>Serum creatinine (mg/dl)<br>Leukocytes (10³/μL)<br>Age (years)<br>Lymphocytes (%)<br>Prothrombin time (seconds)<br>Partial thromboplastin time (seconds)<br>International normalized index (%)<br>Sodium (mmol/L)<br>Magnesium (mg/dL)<br>Potassium (mmol/L)<br>Chlorine (mmol/L)	0.419	0.546	0.27	NT

*The P-value in the analysis is greater than 0.05. NT: no tested
