# Supplementary material for: Inflammatory Metabolic Index and Metabolic-Inflammatory Stress Index as New Biomarkers for Complicated and Perforated Acute Appendicitis
Source: J Clin Med. 2025 Jul 25;14(15):5281. doi: 10.3390/jcm14155281 (PMC12347975; doi:10.3390/jcm14155281)
Supplement: Supplementary file 1 [file jcm-14-05281-s001.zip › 0-Supplementary Table S2.pdf]

Supplementary Table S2. Combinations of variables analyzed (Models) through factor analysis, for surgical diagnosis, and their KMO values.

Model	KMO value			
	Stage I	Stage II	Stage III	Stage IV
**Model 1**				
Platelets (10^3/µL)				
Leukocytes (10^3/µL)				
Lymphocytes (%)				
Neutrophils (%)				
Neutrophil-lymphocyte ratio				
Platelet-lymphocyte ratio				
Bands (10^3/µL)				
Prothrombin time (seconds)	NT	0.442	0.481	0.570
International normalized index (%)				
Partial thromboplastin time (seconds)				
Glucose (mg/dl)				
Urea (mg/dL)				
Serum creatinine (mg/dl)				
Sex				
Age (years)				
**Model 2**				
Lymphocytes (%)				
Neutrophils (%)	NT	0.539	0.506	0.570
Glucose (mg/dl)				
Serum creatinine (mg/dl)				
**Model 3**				
Prothrombin time (seconds)				
International normalized index (%)				
Partial thrombloplastin time (seconds)				
Urea (mg/dL)	NT	0.564	0.600	0.686
Serum creatinine (mg/dL)				
Neutrophils (%)				
Lymphocytes (%)				
Glucose (mg/dL)				
**Model 4**				
Neutrophils				
Glucose (mg/dl)				
Serum creatinine (mg/dl)				
Prothrombin time (seconds)				
Partial thromboplastin time (seconds)				
International normalized index (%)	NT	0.509	0.505	0.613
Potassium (mmol/L)				
Chlorine (mmol/L)				
Sodium (mmol/L)				
Magnesium (mg/dL)				
Leukocytes (10^3/µL)				
Age (years)				
**Model 5**				
Neutrophils (%)				
Glucose (mg/dl)				
Serum creatinine (mg/dl)	NT	0.507	0.584	0.620
Leukocytes (10^3/µL)				
Age (years)				
Platelets (10^3/µL)				
**Model 6**				
Neutrophils (%)				
Glucose (mg/dl)				
Serum creatinine (mg/dl)				
Leukocytes				
Age (years)				
Lymphocytes (%)				
Prothrombin time (seconds)	NT	0.399	0.444	0.611
Partial thromboplastin time (seconds)				
International normalized index (%)				
Sodium (mmol/L)				
Magnesium (mg/dL)				
Potassium (mmol/L)				
Chlorine (mmol/L)				

NT: no tested
