# Supplementary material for: Inflammatory Metabolic Index and Metabolic-Inflammatory Stress Index as New Biomarkers for Complicated and Perforated Acute Appendicitis
Source: J Clin Med. 2025 Jul 25;14(15):5281. doi: 10.3390/jcm14155281 (PMC12347975; doi:10.3390/jcm14155281)
Supplement: Supplementary file 1 [file jcm-14-05281-s001.zip › 0-Supplementary Table S3.pdf]

Supplementary Table S3. Principal component analysis of significant variables associated with histopathological and surgical diagnosis of appendicitis. Initial autovalues (total, variance and accumulated variance) for the Model 3 are shown by appendicitis stage and classified according to the histopathological and surgical diagnosis.

Component	Histopathological diagnosis											
	Edematous			Suppurative			Gangrenous			Perforated		
	Total	Variance (%)	Accumulated variance (%)	Total	Variance (%)	Accumulated variance (%)	Total	Variance (%)	Accumulated variance (%)	Total	Variance (%)	Accumulated variance (%)
1	2.21	27.651	27.651	2.412	30.15	30.15	2.759	34.484	34.484	4.475	55.937	55.937
2	1.82	22.799	50.451	1.467	18.344	48.494	2.584	32.305	66.79	1.78	22.246	**78.183**
3	1.6	19.98	**70.431**	1.382	17.269	**65.763**	1.237	15.457	**82.247**	NC	NC	NC
**Component**	**Surgical diagnosis**											
	**Stage I**			**Stage II**			**Stage III**			**Stage IV**		
	Total	Variance (%)	Accumulated variance (%)	Total	Variance (%)	Accumulated variance (%)	Total	Variance (%)	Accumulated variance (%)	Total	Variance (%)	Accumulated variance (%)
1	NT	NT	NT	2.422	30.281	30.281	2.393	29.908	29.908	2.953	36.908	36.908
2	NT	NT	NT	1.555	19.433	49.715	2.038	25.471	55.379	1.969	24.612	61.52
3	NT	NT	NT	1.444	18.046	**67.760**	1.216	15.204	**70.583**	1.279	15.992	**77.512**

NT: no tested. NC: no component. The accumulated variance for each stage is highlighted in bold.
