# Supplementary material for: Inflammatory Metabolic Index and Metabolic-Inflammatory Stress Index as New Biomarkers for Complicated and Perforated Acute Appendicitis
Source: J Clin Med. 2025 Jul 25;14(15):5281. doi: 10.3390/jcm14155281 (PMC12347975; doi:10.3390/jcm14155281)
Supplement: Supplementary file 1 [file jcm-14-05281-s001.zip › 0-Supplementary Table S4.pdf]

Supplementary Table S4. Rotated Component Matrix (Varimax) for variables associated with histopathological and surgical diagnosis of acute appendicitis: Model 3.

Variables in the Model	Histopathoological diagnosis											
	Component 1				Component 2				Component 3			
	Edematous	Suppurative	Gangrenous	Perforated	Edematous	Suppurative	Gangrenous	Perforated	Edematous	Suppurative	Gangrenous	Perforated
Prothrombin time (seconds)	0.981	0.938	-	0.976	-	-	0.964	-	-	-	-	-
International normalized index (%)	0.970	0.942	-	0.974	-	-	0.943	-	-	-	-	-
Partial thrombloplastin time (seconds)	0.464	0.782	-	0.823	-	-	0.715	-	-	-	-	-
Urea (mg/dL)	-	-	0.882	0.913	0.941	0.800	-	-	-	-	-	-
Serum creatinine (mg/dL)	-	-	0.858	-	0.913	0.503	-	0.676	-	-	-	-
Neutrophils (%)	-	-	-	-	-	-	-	-0.807	-0.737	0.775	0.765	-
Lymphocytes (%)	-	-	-0.527	-	-	-	-	-0.661	0.723	-0.768	-	-
Glucose (mg/dL)	-	-	0.926	0.873	-	0.727	-	-	0.565	-	-	-

Variables in the Model	Surgical diagnosis											
	Component 1				Component 2				Component 3			
	Stage I	Stage II	Stage III	Stage IV	Stage I	Stage II	Stage III	Stage IV	Stage I	Stage II	Stage III	Stage IV
Prothrombin time (seconds)	NT	0.957	0.949	0.956	NT	-	-	-	NT	-	-	-
International normalized index (%)	NT	0.960	0.947	0.957	NT	-	-	-	NT	-	-	-
Partial thrombloplastin time (seconds)	NT	0.600	0.741	0.798	NT	-	-	-	NT	-	-	-
Urea (mg/dL)	NT	-	-	-	NT	-	0.917	0.671	NT	0.673	-	-
Serum creatinine (mg/dL)	NT	-	-	-	NT	-	0.875	0.862	NT	0.773	-	-
Neutrophils (%)	NT	-	-	-	NT	0.663	-	-	NT	-	0.794	0.798
Lymphocytes (%)	NT	-	-	-	NT	-0.767	-	-	NT	-	-0.725	-0.750
Glucose (mg/dL)	NT	-	-	-	NT	0.623	0.555	0.825	NT	-	-	-

The values that were deemed to be of insufficient statistical significance have been omitted from the displayed data set (-). Only those values that were found to be statistically significant have been included. NT: no tested.
